# Supplementary material for: Immune response to SARS-CoV-2 variants of concern in vaccinated individuals
Source: Nat Commun. 2021 May 25;12:3109. doi: 10.1038/s41467-021-23473-6 (PMC8149389; doi:10.1038/s41467-021-23473-6)
Supplement: Supplementary file 1 — Supplementary Information [file 41467_2021_23473_MOESM1_ESM.pdf]

Supplementary Information – Immune response to SARS-CoV-2 variants of concern in vaccinated individuals

Matthias Becker[1#], Alex Dulovic[1#], Daniel Junker[1], Natalia Ruetalo[2], Philipp D. Kaiser[1], Yudi T. Pinilla[3], Constanze Heinzel[3], Julia Haering[1], Bjoern Traenkle[1], Teresa R. Wagner[1,4], Mirjam Layer[2], Martin Mehrlaender[5], Valbona Mirakaj[5], Jana Held[3,6], Hannes Planatscher[7], Katja Schenke-Layland[1,8,9,10], Gérard Krause[11,12], Monika Strengert[11,12], Tamam Bakchoul[13], Karina Althaus[13], Rolf Fendel[3,6], Andrea Kreidenweiss[3,6], Michael Koeppen[5], Ulrich Rothbauer[1,4*], Michael Schindler[2*], Nicole Schneiderhan-Marra[1*]

Author Affiliations

1 NMI Natural and Medical Sciences Institute at the University of Tübingen, Reutlingen, Germany

2 Institute for Medical Virology and Epidemiology, University Hospital Tübingen, Tübingen, Germany

3 Institute of Tropical Medicine, University of Tübingen, Germany

4 Pharmaceutical Biotechnology, University of Tübingen, Germany

5 Department of Anaesthesiology and Intensive Care Medicine, University Hospital Tübingen, Tübingen, Germany

6 German Center for Infection Research (DZIF), partner site Tübingen, Germany

7 Signatope GmbH, Reutlingen, Germany

8 Cluster of Excellence iFIT (EXC2180) "Image-Guided and Functionally Instructed Tumor Therapies", University of Tübingen, Tübingen, Germany

9 Department of Women's Health, Research Institute for Women's Health, University of Tübingen, Tübingen, Germany

10 Department of Medicine/Cardiology, Cardiovascular Research Laboratories, David Geffen School of Medicine at UCLA, Los Angeles, USA

11 Helmholtz Centre for Infection Research, Braunschweig, Germany

12 TWINCORE GmbH, Centre for Experimental and Clinical Infection Research, a joint venture of the Hannover Medical School and the Helmholtz Centre for Infection Research, Hannover, Germany

13 Institute for Clinical and Experimental Transfusion Medicine, University Hospital Tübingen, Tübingen, Germany

# these authors contributed equally to this work

* corresponding authors.

Contact Information

Nicole Schneiderhan-Marra – Phone number +49 (0)7121 51530 815. Email Address Nicole.schneiderhan@nmi.de Postal Address – Markwiesenstrasse 55, 72770 Reutlingen, Germany.

Michael Schindler – Phone number +49 (0)7071 2987459. Email Address Michael.Schindler@med.uni-tuebingen.de Postal Address – Elfriede-Aulhorn-Strasse 6, 72076 Tübingen, Germany

Ulrich Rothbauer – Phone number +49 (0)7121 51530 415. Email Address Ulrich.rothbauer@nmi.de Postal Address – Markwiesenstrasse 55, 72770 Reutlingen, Germany

Competing Interests

T.R.W., P.K., N.S.M. and U.R. are named as inventors on a patent application (EP 20 197 031.6) claiming the use of the described Nanobodies used in the NeutrobodyPlex for diagnosis and therapeutics filed by the Natural and Medical Sciences Institute. The other authors declare no competing interest.

Supplementary Table 1 – Characteristics of Vaccinated and Infected sera

Characteristic	Vaccinated	Infected
Number of donors	23	35
Median age (IQR) – years	42 (16)	59 (19)
Female sex (%)	5 (21.7)	10 (28.6)
1st sample collection – median ΔT post first vaccination (range)	21 days (-1 to 22 days)	Not applicable
2nd sample collection – median ΔT post second vaccination (range)	15 days (-7 to 16 days)	Not applicable
Median ΔT post positive PCR test (range)	Not applicable	14 days (2 – 85 days)

For both vaccinated and infected groups, the number of donors, median age in years (including inter-quartile range) and number of females are provided. For vaccinated groups, the median time post-first vaccination is provided for the first sample, and the median time post-second vaccination is provided for the second sample. The full range is included for both samples. A total of 45 samples from 23 vaccinated donors were included in this study. For one individual a second sample was not taken. One individual had their first sample collected before they received a vaccination and so they have a negative ΔT for the first sample. Similarly, two individuals had their second sample collected before they received their second vaccination and so they have a negative ΔT for the second sample. For the infected group, the median ΔT for time between positive PCR test and sample collection is given including the full range.

Supplementary Table 2 – Characteristics of Vaccinated, Infected and Negative saliva samples

Characteristic	Vaccinated	Infected	Negative
Number of donors	22	27	49
Median age (IQR) – years	43 (16)	38 (25 – 58)	29 (25 – 38)
Female sex (%)	5 (22.7)	17 (63)	27 (55)

For all three groups, the total number of samples, median age in years (including inter-quartile range) and number of females are provided. The vaccinated samples are from the same sample group as the serum samples. Infected samples were confirmed by either PCR test or ELISA (EuroImmun) plus the presence of at least one identifying COVID-19 symptom. Negative samples were confirmed to be negative by ELISA (EuroImmun).

Supplementary Table 3 – Full list of primers used in this study

Primer Name	Sequence (5' to 3')
RBDfor	ATATCTAGAGCCACCATGTTCGTGTTTCTGG
N501Yrev	CCACGCCATATGTGGGCTGAAAGCCGTAG
N501Yfor	GGCTTTCAGCCCACATATGGCGTGGGCTATCAGC
RBDrev	AAGATCTGCTAGCTCGAGTCGC
K417Nrev	GTTGTAGTCGGCGATGTTGCCTGTCTGTCCAGGG
K417Nfor	GACAGACAGGCAACATCGCCGACTACAACTACAAGC
E484Krev	GCAGTTGAAGCCTTTCACGCCGTTACAAGGGGT
E484Kfor	GTAACGGCGTGAAAGGCTTCAACTGCTACTTCCC
L452Rrev	CGGTACCGGTAATTGTAGTTGCCGCCG
L452Rfor	GGCAACTACAATTACCGGTACCGGCTGTTCCGGAAG

Full List of primers used in this study for the production of RBD Mutants

Supplementary Table 4 – Antigens included in MULTICOV-AB

Disease	Antigen	Manufacturer	Category number
SARS-CoV-2	Spike Trimer	NMI	-
SARS-CoV-2	RBD	NMI	-
SARS-CoV-2	S1 domain	NMI	-
SARS-CoV-2	S2 domain	Sino	40590
SARS-CoV-2	Nucleocapsid	Aalto	6404-b
SARS-CoV-2	Nucleocapsid N-terminal domain	NMI	-
hCoV-OC43	Spike	Sino	40607-V08B
hCoV-OC43	S1 domain	NMI	-
hCoV-OC43	Nucleocapsid	NMI	-
hCoV-OC43	Nucleocapsid N-terminal domain	NMI	-
hCoV-HKU1	S1 domain	NMI	-
hCoV-HKU1	Nucleocapsid	NMI	-
hCoV-HKU1	Nucleocapsid N-terminal domain	NMI	-
hCoV-NL63	Spike Trimer	NMI	-
hCoV-NL63	S1 domain	NMI	-
hCoV-NL63	Nucleocapsid	NMI	-
hCoV-NL63	Nucleocapsid N-terminal domain	NMI	-
hCoV-229E	S1 domain	NMI	-
hCoV-229E	Nucleocapsid	NMI	-
hCoV-229E	Nucleocapsid N-terminal domain	NMI	-

Full list of antigens included as standard in MULTICOV-AB (minus controls), their manufacturer, and if available, their category number. Full details on all NMI produced antigens can be found in [1].

Supplementary Figure 1 – High serum antibody titers after the second vaccination dose

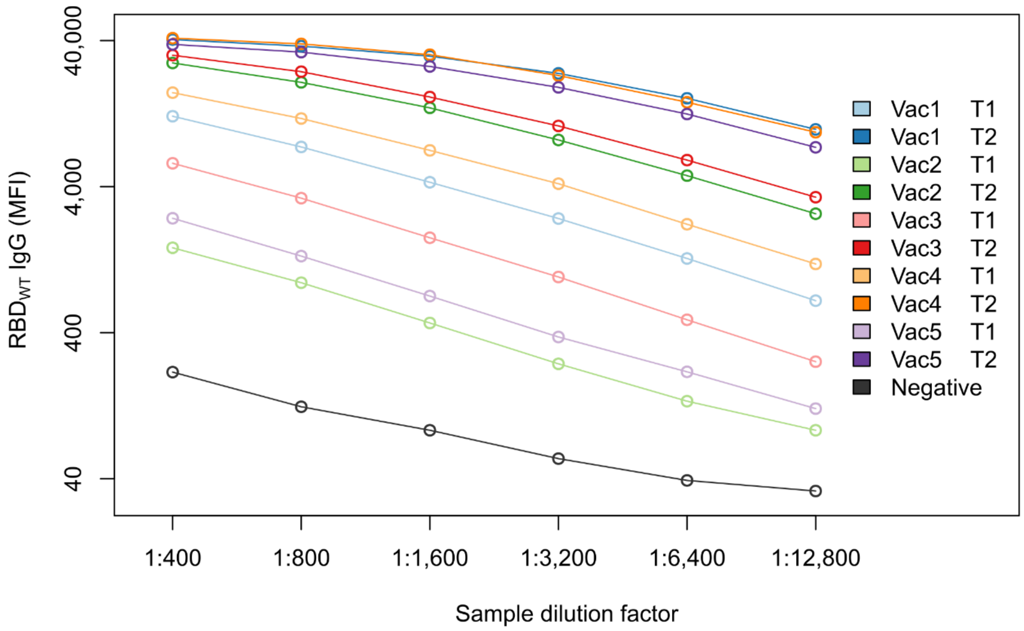

The second vaccination appears to plateau the serum antibody response at the Upper Limit of Detection for MULTICOV-AB. To confirm this, 11 samples consisting of 10 paired samples from five vaccinated individuals (shown in paired colours) and one negative individual (shown in black) were examined in a dilution series. Due to the wide range of samples, a log curve is used for the y axis. The three sera with the highest response maintained a similarly high response for the initial dilution, indicating a plateau in the range of >40,000 MFI, therefore suggesting that even for donors with high responses in the first sample, the second vaccination strongly increased the antibody response. A uniform curve shape for all samples (including the negative control) confirmed reliability of the generated data. Source data are provided as a Source Data file.

Supplementary Figure 2 – RBD reactive IgG detected by ELISA.

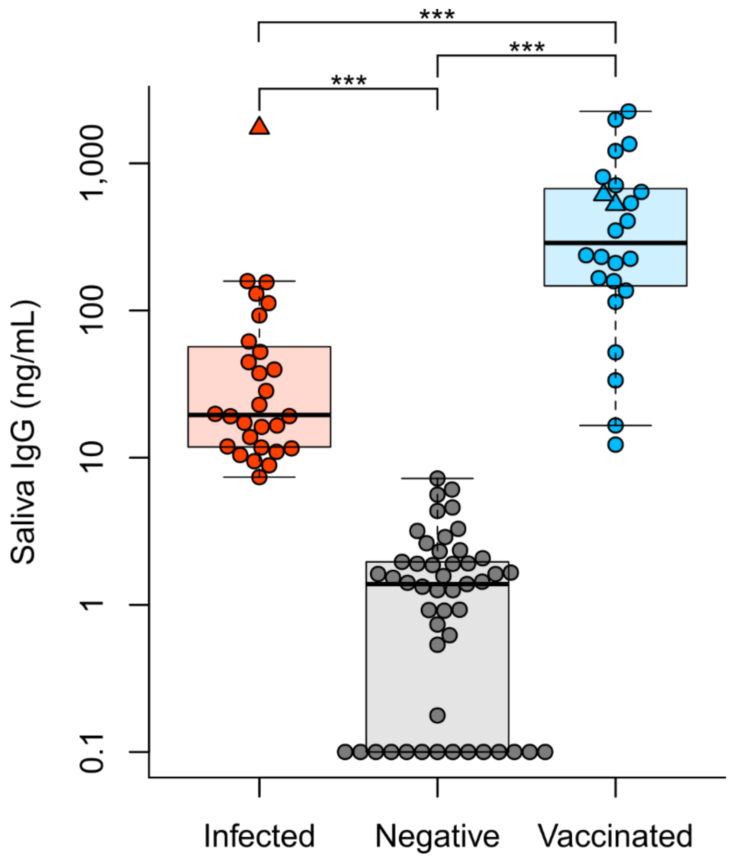

To confirm results for MULTICOV-AB, anti-RBD IgG antibodies in saliva were measured by an in-house ELISA. As in Fig 2, saliva IgG response was highest in vaccinated individuals. Results for vaccinated (blue, n=22), infected (red, n=26) and negative (grey, n=45) individuals are displayed as a Box and whisker plot. Two vaccinated sera samples from individuals not in contact with active SARS-CoV-2 infected individuals and one sera sample from an individual who was previously infected with SARS-CoV-2 and then later vaccinated are included as triangles. Boxes represent the median, 25th and 75th percentiles, whiskers show the largest and smallest non-outlier values. Outliers were determined by 1.5 times IQR. Negative samples that measured 0 were raised to 0.1 for display purposes only. For statistical analysis, their true value was used. Mann-Whitney U (two-sided) was used to determine statistical significance between the groups. *** indicates p-values lower than 0.0001. Source data are provided as a Source Data file.

Supplementary Figure 3 – Cross-reactivity of antibodies to endemic coronaviruses in vaccinated individuals

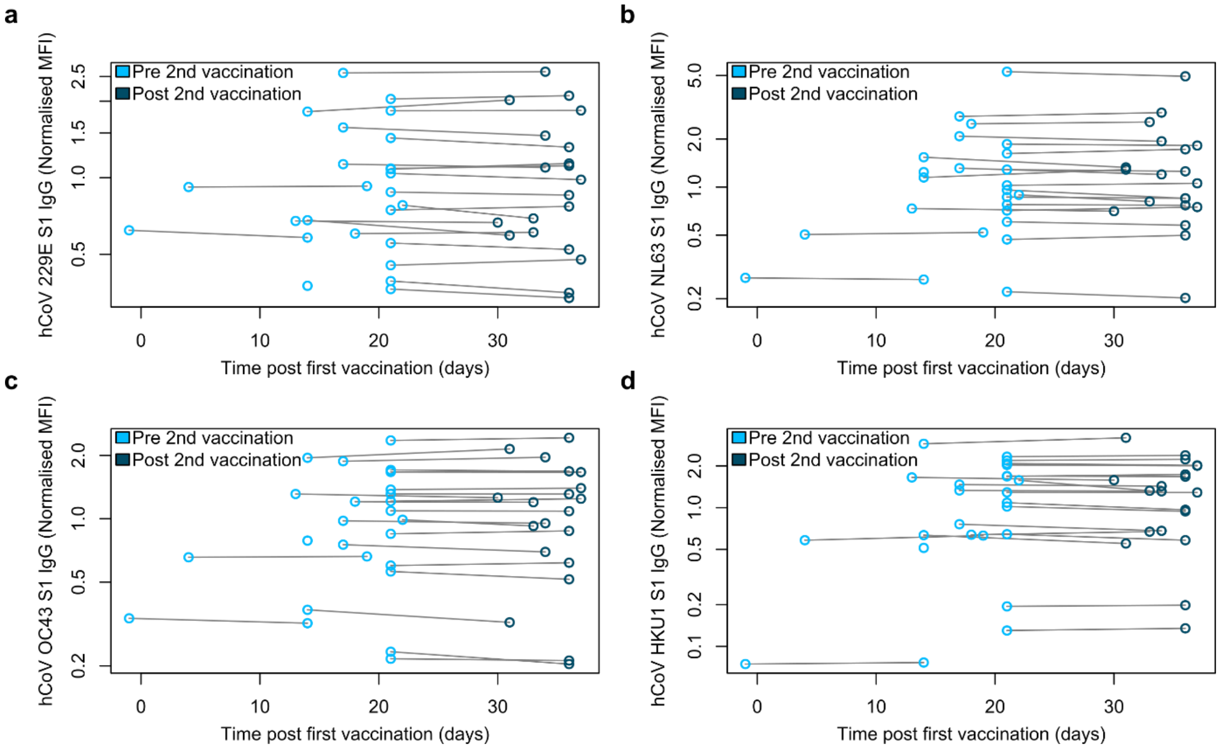

Vaccinated individuals did not have an increased antibody response towards S1 proteins of the endemic coronaviruses 229E (**a**), NL63 (**b**), OC43 (**c**) and HKU1 (**d**). All samples were measured using MULTICOV-AB. Light blue (n=25) indicates samples are pre second vaccination, while dark blue (n=20) indicates samples are post second vaccination. Lines indicated paired samples from the same donor. Source data are provided as a Source Data file.

Supplementary Figure 4 – RBD mutants for the LA and Mink variants have similar antibody binding compared to the wild-type variant.

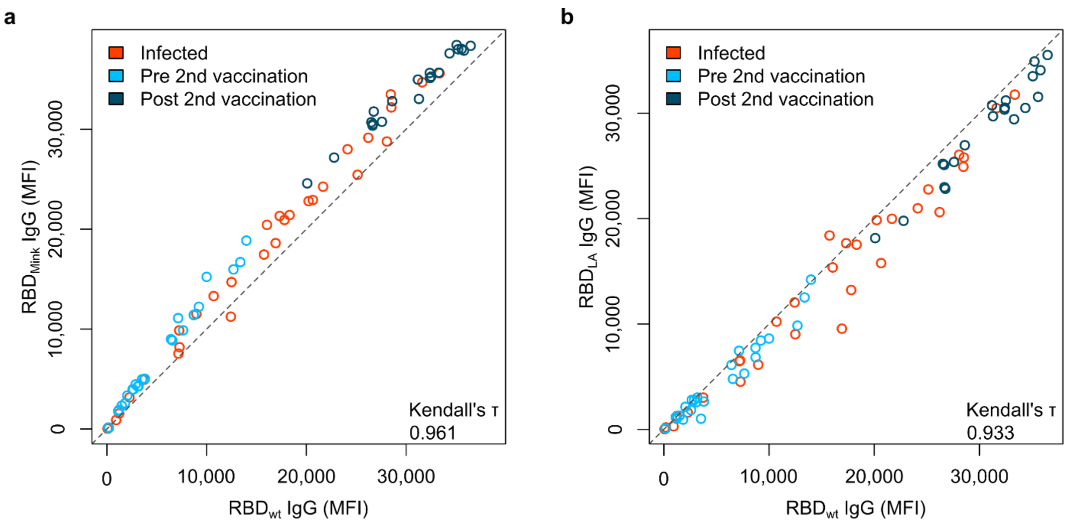

When compared to wild-type (wt), RBD mutants for both the Mink (**a**) and LA (**b**) variants of concern resulted in similar response. RBD mutant antigens were generated (LA) or purchased (Mink) and added to MULTICOV-AB to measure the immune response towards them from vaccinated (blue, n=45) and infected (red, n=35) sera, compared to the wild-type RBD. A linear curve (y=x) is shown as a dashed grey-line to indicate identical response between wild-type and mutant. Kendall's tau was calculated to measure ordinal association between the mutant and wild-type. Source data are provided as a Source Data file.

Supplementary Figure 5 – NeutrobodyPlex confirms reduced neutralization potential of vaccinated and infected sera for the wild-type variant

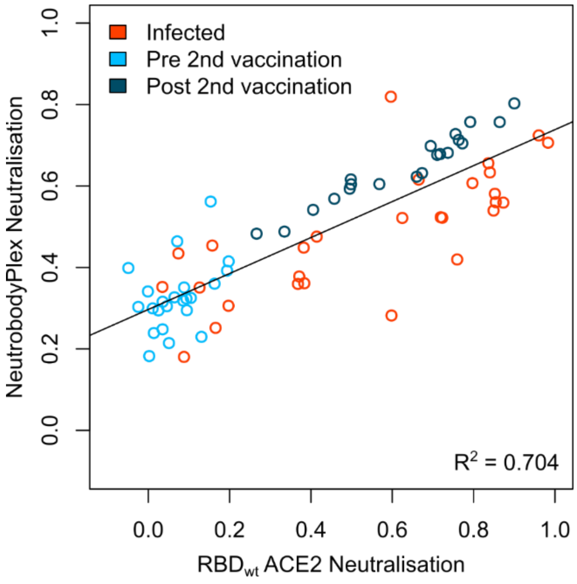

To further validate the results of the VNT and ACE2 competition assay, NeutrobodyPlex was used to examine neutralization potential of sera from vaccinated (pre-second dose (light blue, n=23), post-second dose (dark blue, n=20)) and infected individuals (red, n=28) for wild-type (wt) RBD. The correlation between NeutrobodyPlex and ACE2 competition assay results is shown. A linear regression (y=x) was calculated with the R^2 value shown. Source data are provided as a Source Data file.

Supplementary Figure 6 – Neutralization as measured by ACE2 inhibition assay for the UK, Mink and LA RBD mutants compared to wild-type RBD

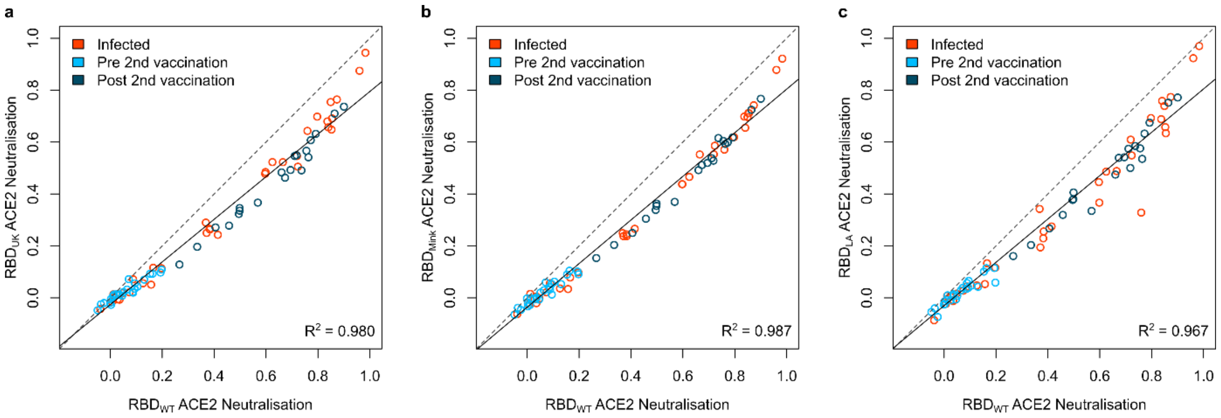

To determine the effect variants of concern had upon neutralization potential, an ACE2 competition assay was developed. RBD mutants for all variants of concern included within this manuscript (UK (**a**), Mink (**b**), LA (**c**)) were examined as well as the wild-type (wt) variant on sera from infected (red, n=35) and vaccinated (pre second vaccination (light blue, n=25), post second vaccination (dark blue, n=20)) individuals. Linear regression (y=x) was calculated for each panel, with the R^2 value shown. Linear regressions had the following equations for the different figure panels: (**a**) y = -0.026 + 0.820x (**b**) y = -0.036 + 0.840x (**c**) y = -0.03 + 0.835x. Source data are provided as a Source Data file.

Supplementary Figure 7 – Overview of VNT

a

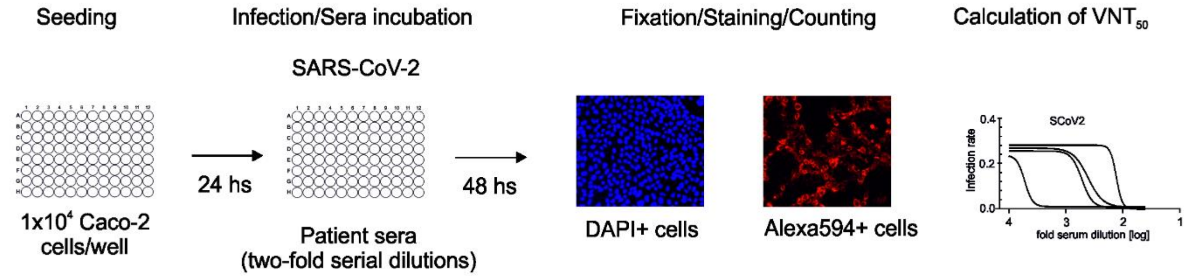

b

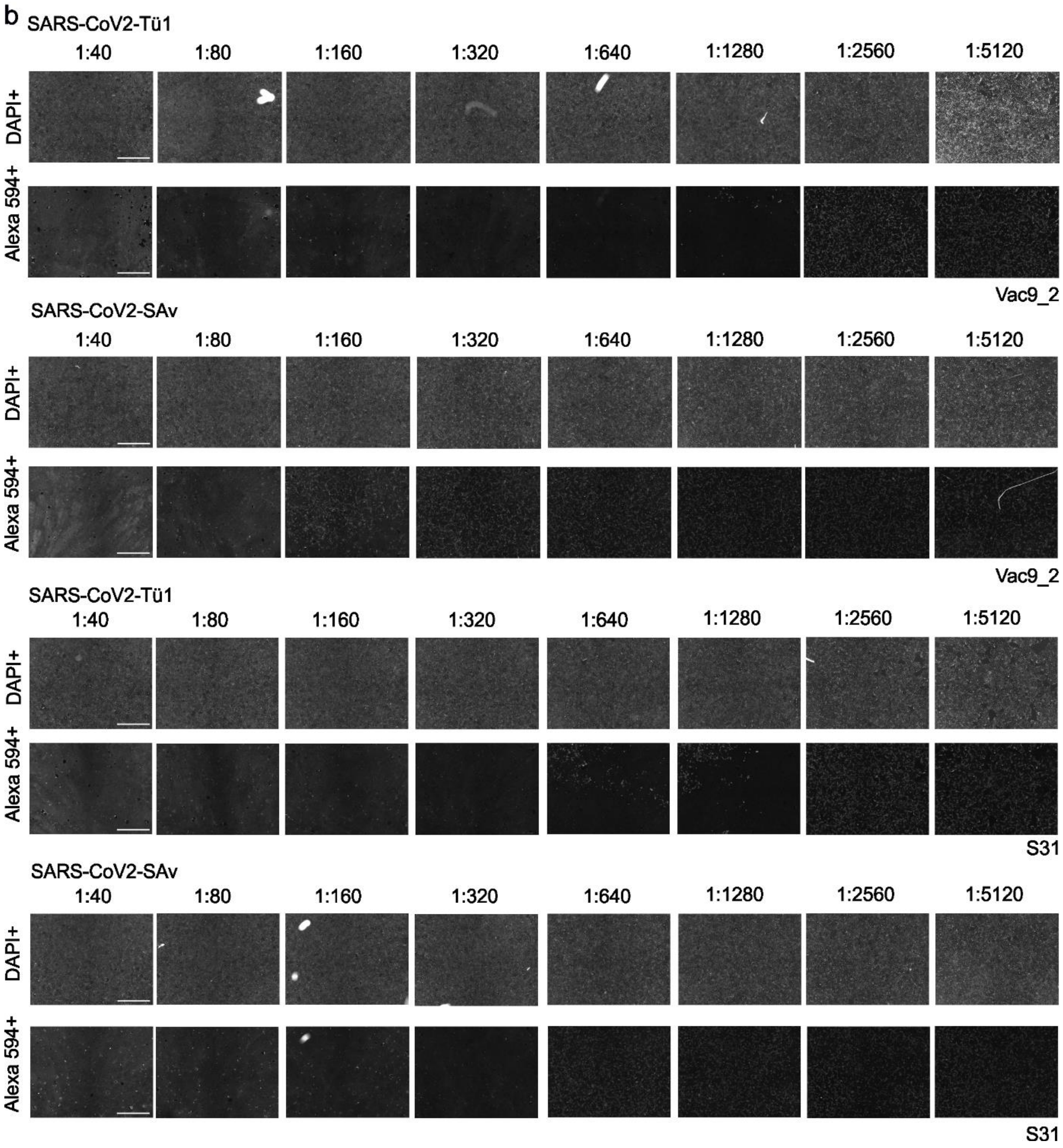

Simple overview of the VNT protocol (**a**) with examples (**b**) for cells treated with the wild-type (SARS-CoV-2-Tü1) and South African (SARS-CoV-2-SAv) for sera from one vaccinated (Vac9_2) and one infected (S31) individual. A dilution series (1:40 – 1:5120) is shown with the corresponding images for DAPI-positive and Alexa 594-positive cells. Scale bar = 1000 µm.

References

1 Becker, M. *et al.* Exploring beyond clinical routine SARS-CoV-2 serology using MultiCoV-Ab to evaluate endemic coronavirus cross-reactivity. *Nature Communications* **12**, 1152, doi:10.1038/s41467-021-20973-3 (2021).
