# Supplementary material for: Immune response to SARS-CoV-2 variants of concern in vaccinated individuals
Source: Nat Commun. 2021 May 25;12:3109. doi: 10.1038/s41467-021-23473-6 (PMC8149389; doi:10.1038/s41467-021-23473-6)
Supplement: Supplementary file 3 — Description of Additional Supplementary Files [file 41467_2021_23473_MOESM3_ESM.pdf]

Description of Additional Supplementary Files

File Name: Supplementary Data 1
Description: **Alignment of Spike Protein for Tübingen Isolate 200325_Tü1 and South Africa Isolate 210211_SAv** A sequence alignment of the Spike proteins used in the virus neutralization test is shown. Amino acids differing from Tü1 are denoted in the SA isolate and the consensus sequence.
