# Supplementary material for: Immune response to SARS-CoV-2 variants of concern in vaccinated individuals
Source: Nat Commun. 2021 May 25;12:3109. doi: 10.1038/s41467-021-23473-6 (PMC8149389; doi:10.1038/s41467-021-23473-6)
Supplement: Supplementary file 4 — Supplementary Data 1 [file 41467_2021_23473_MOESM4_ESM.pdf]

Supplementary Data 1 – Alignment of Spike Protein for Tübingen Isolate 200325_Tü1 and South Africa Isolate 210211_SAv

```
      Tü1  MFVFLVLLPL VSSQCVNLTT RTQLPPAYTN SFTRGVYYPD KVFRSSVLHS TQDLFLPFFS NVTWFHAIHV
       SA  .......... .......F.. .......... .......... .......... .......... ..........
Consensus  .......... .......f.. .......... .......... .......... .......... ..........

           71                                                                    140
      Tü1  SGTNGTKRFD NPVLPFNDGV YFASTEKSNI IRGWIFGTTL DSKTQSLLIV NNATNVVIKV CEFQFCNDPF
       SA  .........A .......... .......... .......... .......... .......... ..........
Consensus  .........a .......... .......... .......... .......... .......... ..........

           141                                                                   210
      Tü1  LGVYYHKNNK SWMESEFRVY SSANNCTFEY VSQPFLMDLE GKQGNFKNLR EFVFKNIDGY FKIYSKHTPI
       SA  .......... .......... .......... .......... .......... .......... ..........
Consensus  .......... .......... .......... .......... .......... .......... ..........

           211                                                                   280
      Tü1  NLVRDLPQGF SALEPLVDLP IGINITRFQT LLALHRSYLT PGDSSSGWTA GAAAYYVGYL QPRTFLLKYN
       SA  ....G..... .......... .......... .......... .......... .......... ..........
Consensus  ....d..... .......... .......... .......... .......... .......... ..........

           281                                                                   350
      Tü1  ENGTITDAVD CALDPLSETK CTLKSFTVEK GIYQTSNFRV QPTESIVRFP NITNLCPFGE VFNATRFASV
       SA  .......... .......... .......... .......... .......... .......... ..........
Consensus  .......... .......... .......... .......... .......... .......... ..........

           351                                                                   420
      Tü1  YAWNRKRISN CVADYSVLYN SASFSTFKCY GVSPTKLNDL CFTNVYADSF VIRGDEVRQI APGQTGKIAD
       SA  .......... .......... .......... .......... .......... .......... ......N...
Consensus  .......... .......... .......... .......... .......... .......... ......k...

           421                                                                   490
      Tü1  YNYKLPDDFT GCVIAWNSNN LDSKVGGNYN YLYRLFRKSN LKPFERDIST EIYQAGSTPC NGVEGFNCYF
       SA  .......... .......... .......... .......... .......... .......... ...K......
Consensus  .......... .......... .......... .......... .......... .......... ...e......

           491                                                                   560
      Tü1  PLQSYGFQPT NGVGYQPYRV VVLSFELLHA PATVCGPKKS TNLVKNKCVN FNFNGLTGTG VLTESNKKFL
       SA  .......... Y......... .......... .......... .......... .......... ..........
```

```
Consensus  .......... n......... .......... .......... .......... .......... ..........

           561                                                                      630
      Tü1  PFQQFGRDIA DTTDAVRDPQ TLEILDITPC SFGGVSVITP GTNTSNQVAV LYQGVNCTEV PVAIHADQLT
       SA  .......... .......... .......... .......... .......... .......... ..........
Consensus  .......... .......... .......... .......... .......... .......... ..........

           631                                                                      700
      Tü1  PTWRVYSTGS NVFQTRAGCL IGAEHVNNSY ECDIPIGAGI CASYQTQTNS PRRARSVASQ SIIAYTMSLG
       SA  .......... .......... .......... .......... .......... .......... ..........
Consensus  .......... .......... .......... .......... .......... .......... ..........

           701                                                                      770
      Tü1  AENSVAYSNN SIAIPTNFTI SVTTEILPVS MTKTSVDCTM YICGDSTECS NLLLQYGSFC TQLNRALTGI
       SA  V......... .......... .......... .......... .......... .......... ..........
Consensus  a......... .......... .......... .......... .......... .......... ..........

           771                                                                      840
      Tü1  AVEQDKNTQE VFAQVKQIYK TPPIKDFGGF NFSQILPDPS KPSKRSFIED LLFNKVTLAD AGFIKQYGDC
       SA  .......... .......... .......... .......... .......... .......... ..........
Consensus  .......... .......... .......... .......... .......... .......... ..........

           841                                                                      910
      Tü1  LGDIAARDLI CAQKFNGLTV LPPLLTDEMI AQYTSALLAG TITSGWTFGA GAALQIPFAM QMAYRFNGIG
       SA  .......... .......... .......... .......... .......... .......... ..........
Consensus  .......... .......... .......... .......... .......... .......... ..........

           911                                                                      980
      Tü1  VTQNVLYENQ KLIANQFNSA IGKIQDSLSS TASALGKLQD VVNQNAQALN TLVKQLSSNF GAISSVLNDI
       SA  .......... .......... .......... .......... .......... .......... ..........
Consensus  .......... .......... .......... .......... .......... .......... ..........

           981                                                                     1050
      Tü1  LSRLDKVEAE VQIDRLITGR LQSLQTYVTQ QLIRAAEIRA SANLAATKMS ECVLGQSKRV DFCGKGYHLM
       SA  .......... .......... .......... .......... .......... .......... ..........
Consensus  .......... .......... .......... .......... .......... .......... ..........

           1051                                                                    1120
      Tü1  SFPQSAPHGV VFLHVTYVPA QEKNFTTAPA ICHDGKAHFP REGVFVSNGT HWFVTQRNFY EPQIITTDNT
       SA  .......... .......... .......... .......... .......... .......... ..........
Consensus  .......... .......... .......... .......... .......... .......... ..........
```

```
           1121                                                                    1190
      Tü1  FVSGNCDVVI GIVNNTVYDP LQPELDSFKE ELDKYFKNHT SPDVDLGDIS GINASVVNIQ KEIDRLNEVA
       SA  .......... .......... .......... .......... .......... .......... ..........
Consensus  .......... .......... .......... .......... .......... .......... ..........

           1191                                                                    1260
      Tü1  KNLNESLIDL QELGKYEQYI KWPWYIWLGF IAGLIAIVMV TIMLCCMTSC CSCLKGCCSC GSCCKFDEDD
       SA  .......... .......... .......... .......... .......... .......... ..........
Consensus  .......... .......... .......... .......... .......... .......... ..........

           1261      1273
      Tü1  SEPVLKGVKL HYT
       SA  .......... ...
Consensus  .......... ...
```
