# Supplementary material for: Immune response to SARS-CoV-2 variants of concern in vaccinated individuals
Source: Nat Commun. 2021 May 25;12:3109. doi: 10.1038/s41467-021-23473-6 (PMC8149389; doi:10.1038/s41467-021-23473-6)
Supplement: Supplementary file 5 — Reporting Summary [file 41467_2021_23473_MOESM5_ESM.pdf]

nature research

Corresponding author(s): Nicole Schneiderhan-Marra, Michael Schindler, Ulrich Rothbauer

Last updated by author(s): Apr 23, 2021

Reporting Summary

Nature Research wishes to improve the reproducibility of the work that we publish. This form provides structure for consistency and transparency in reporting. For further information on Nature Research policies, see our Editorial Policies and the Editorial Policy Checklist.

Statistics

For all statistical analyses, confirm that the following items are present in the figure legend, table legend, main text, or Methods section.

n/a	Confirmed	
☐	☒	The exact sample size (*n*) for each experimental group/condition, given as a discrete number and unit of measurement
☐	☒	A statement on whether measurements were taken from distinct samples or whether the same sample was measured repeatedly
☐	☒	The statistical test(s) used AND whether they are one- or two-sided<br>*Only common tests should be described solely by name; describe more complex techniques in the Methods section.*
☒	☐	A description of all covariates tested
☒	☐	A description of any assumptions or corrections, such as tests of normality and adjustment for multiple comparisons
☐	☒	A full description of the statistical parameters including central tendency (e.g. means) or other basic estimates (e.g. regression coefficient) AND variation (e.g. standard deviation) or associated estimates of uncertainty (e.g. confidence intervals)
☐	☒	For null hypothesis testing, the test statistic (e.g. *F*, *t*, *r*) with confidence intervals, effect sizes, degrees of freedom and *P* value noted<br>*Give P values as exact values whenever suitable.*
☒	☐	For Bayesian analysis, information on the choice of priors and Markov chain Monte Carlo settings
☒	☐	For hierarchical and complex designs, identification of the appropriate level for tests and full reporting of outcomes
☒	☐	Estimates of effect sizes (e.g. Cohen's *d*, Pearson's *r*), indicating how they were calculated

Our web collection on statistics for biologists contains articles on many of the points above.

Software and code

Policy information about availability of computer code

Data collection	Data was collected using the following instruments and softwares:<br>Luminex FLEXMAP 3D instrument with xPONENT Software 4.3,<br>Biotek Cytation 3 with BioTek Gen5 Software,<br>CLARIOstar with Clariostar Software Version 5.40 R2 and MARS Data Analysis Software Version 3.31
Data analysis	Data analysis and figure generation was performed in RStudio (Version 1.2.5001), running R (version 3.6.1) with the additional packages “beeswarm” and “RcolorBrewer” for data depiction purposes only. The type of statistical analysis performed (when appropriate) is listed in the figure legends. Figures were generated in Rstudio and then edited for clarity in Inkscape (Inkscape 0.92.4). Mann-Whitney U test was used to determine difference between signal distributions from different sample groups using the “wilcox.test” function from R’s “stats” library. Kendall’s τ coefficient was calculated in order to determine ordinal association between the observed antibody responses towards RBD mutant and wild-type proteins using the “cor” function from R’s “stats” library. Linear regression was performed to assess reduction in ACE2 neutralization observed for RBD mutants compared to wild-type proteins using the “lm” function from R’s “stats” library. Pre-processing of data such as matching sample metadata and collecting results from multiple assay runs was performed in Excel 2016. GraphPad Prism version 8.4.0 was used to process VNT data. Custom analysis code in R and required input files have been deposited on GitHub: https://github.com/BeckerMatthias/Vaccination_VoC_Publication

For manuscripts utilizing custom algorithms or software that are central to the research but not yet described in published literature, software must be made available to editors and reviewers. We strongly encourage code deposition in a community repository (e.g. GitHub). See the Nature Research guidelines for submitting code & software for further information.

Data

Policy information about availability of data

All manuscripts must include a data availability statement. This statement should provide the following information, where applicable:

- Accession codes, unique identifiers, or web links for publicly available datasets
- A list of figures that have associated raw data
- A description of any restrictions on data availability

Source data are provided with this paper.

Field-specific reporting

Please select the one below that is the best fit for your research. If you are not sure, read the appropriate sections before making your selection.

[x] Life sciences [ ] Behavioural & social sciences [ ] Ecological, evolutionary & environmental sciences

For a reference copy of the document with all sections, see nature.com/documents/nr-reporting-summary-flat.pdf

Life sciences study design

All studies must disclose on these points even when the disclosure is negative.

Sample size	Sample size was based on maximal available samples from vaccinated individuals with two time points per individual where possible and samples of infected and uninfected individuals with one time point per individual.
Data exclusions	No Data was excluded from analysis.
Replication	Performance of MULTICOV-AB and NeutrobodyPlex was verified with quality control samples processed on every plate. As a result, these experiments were not replicated. For Saliva and ACE2 measurements appropriate controls were processed on each plate and all samples were measured in duplicates. Saliva IgG ELISA performance was determined using a standard cuve processed on each plate and measurements were not replicated. VNT assay was replicated for a subset of sera two times to ensure data reproducibility, furthermore a previously titered control sera was included as internal standards. Replication when performed was successful.
Randomization	No randomization steps were performed for experiments or data analysis. For measurements, samples from all three groups (vaccinated, infected, negative) were included on each plate to further eliminate bias. Samples were analyzed and grouped based on available metadata on vaccination and infection timepoints and sample drawing timepoints. For the performed analyses no randomization was required. A subset of samples was manually selected for the virus neutralisation test, in order to best cover the full range of observed reactivities in MULTICOV-AB.
Blinding	Blinding was not available for this study due to sample collection. As there was no associated therapeutic intervention, and in line with our randomization plan, we chose not to perform blinding on sample measurements.

Reporting for specific materials, systems and methods

We require information from authors about some types of materials, experimental systems and methods used in many studies. Here, indicate whether each material, system or method listed is relevant to your study. If you are not sure if a list item applies to your research, read the appropriate section before selecting a response.

Materials & experimental systems

n/a	Involved in the study	
[ ]	[x]	Antibodies
[ ]	[x]	Eukaryotic cell lines
[x]	[ ]	Palaeontology and archaeology
[x]	[ ]	Animals and other organisms
[ ]	[x]	Human research participants
[x]	[ ]	Clinical data
[x]	[ ]	Dual use research of concern

Methods

n/a	Involved in the study	
[x]	[ ]	ChIP-seq
[x]	[ ]	Flow cytometry
[x]	[ ]	MRI-based neuroimaging

Antibodies

Antibodies used	Antibodies bound to microspheres were detected with R-phycoerythrin labeled goat-anti-human IgG or IgA antibodies:<br>goat-anti-human-IgG-RPE (Dianova, Cat# 109-116-098, Lot#149288)<br>goat-anti-human-IgA-RPE (Dianova, Cat# 109-115-011, Lot#149254)<br>biotinylated anti-human IgG (#109-065-008, Jackson Immuno Research Laboratories)
Validation	Both RPE-labelled detection antibodies used in the developed assays are widely used in assays based on xMAP Technology and are Immunoglobulin subtype specific as stated on the manufacturers website. We have further cross-validated the antibodies by measuring backgound signals when incubating with microshperes coated with human IgA or human IgG and did not find notable immunoglobulin-species cross-reactivity (data not shown).<br>The biotinylated anti-human IgG (#109-065-008, Jackson Immuno Research Laboratories) is a commercially available antibody which has been previously used in publications as per the manufacturers website (https://www.jacksonimmuno.com/catalog/products/109-065-008)

Eukaryotic cell lines

Policy information about cell lines

Cell line source(s)	Expi293 were sourced from ThermoFisher.<br>Caco-2 (Human Colorectal adenocarcinoma, ATCC#HTB-37 or ECACC#09042001) were kindly gifted from the Interfaculty Institute for Microbiology and Infection Medicine Tübingen (IMIT), Prof. Julia Frick.
Authentication	Expi293 were commercially purchased and no authentication was performed.<br>No authentication was performed for Caco-2 cells.
Mycoplasma contamination	Expi293 cell line used for protein expression were not tested for mycoplasma contamination.<br>Caco-2 cell line was regularily tested negative for mycoplasma using the PCR mycoplasma kit Venor GeM Classic (Minerva Biolabs, cat. #11-1025) and the Taq DNA Polymerase (Minerva Biolabs, cat. #53-0100)
Commonly misidentified lines<br>(See ICLAC register)	No commonly misidentified cell lines were used in this study.

Human research participants

Policy information about studies involving human research participants

Population characteristics	Serum and saliva samples were collected from healthcare workers, vaccinated with the Pfizer BNT-162b2 vaccine. Two serum samples were collected from each individual. The first collection took place on average 17 days following administration of the first dose, while the second collection took place on average 12 days following administration of the second dose. Saliva samples were collected 7-10 days following administration of the second dose. Infected serum and saliva samples were collected from different groups of individuals. Serum samples were collected from individuals hospitalized at Universität Klinikum Tübingen between 25.03.2020 and 22.01.2021. All individuals tested positive for SARS-CoV-2 by PCR. Saliva samples were collected from individuals who had previously been infected with SARS-CoV-2 between 07.03.2020 and 19.06.2020. All individuals had previously tested positive by PCR, or were confirmed as being previously infected by ELISA measurements plus the presence of at least one key symptom (i.e. Coughing, Fever). Negative serum and saliva samples were also from different groups of individuals. Negative serum samples were purchased from Central Biohub. All of these samples were collected pre-pandemic. Negative saliva samples were collected at the Institute of Tropical Medicine, Universität Tübingen from negative individuals. As additional controls, serum and saliva samples were collected from two individuals vaccinated with Pfizer BNT-162b2, who do not have contact with active SARS-CoV-2 infected patients, and one individual who had been previously infected with SARS-CoV-2 and was later vaccinated.<br>An overview of the study population is also given in Supplementary Tables 1 and 2.
Recruitment	Due to when the study was devised and the samples were collected, in accordance with the german national vaccine strategy for SARS-CoV-2, all vaccinated donors were healthcare workers at assorted clinics of the University of Tübingen Hospitals. Healthcare workers came from the same department and therefore are of mixed age and gender and have an equal previous exposure to SARS-CoV-2. As a control, 2 vaccinated donors are individuals who do not have contact with active COVID-19 patients. Infected and non-infected donors were not recruited explicitly for this study and cannot be considered biased.
Ethics oversight	This study was approved by the Ethics Committee of Eberhard Karls University Tübingen and the University Hospital Tübingen under the approval number 222/2020BO2 to Dr. Karina Althaus, Institute for Clinical and Experimental Transfusion Medicine, University Hospital Tübingen, and 312/2020BO1 (Coro-Buddy) to Dr. Andrea Kreidenweiss, Institute for Tropical Medicine, University Hospital Tübingen and Eberhard Karls University Tübingen. All sample donors gave written informed consent.

Note that full information on the approval of the study protocol must also be provided in the manuscript.
